# Use of Simulation in Canadian Neonatal-Perinatal Medicine Training Programs

**DOI:** 10.7759/cureus.1448

**Published:** 2017-07-08

**Authors:** Jonathan Wong, Emer Finan, Douglas Campbell

**Affiliations:** 1 Pediatrics, University of British Columbia; 2 Pediatrics, Mt. Sinai Health Systems; 3 Pediatrics, St. Michael's Hospital, University of Toronto

**Keywords:** simulation based medical education, survey, neonatal training, procedural training

## Abstract

Introduction

Simulation is used for the delivery of education and on occasion assessment. Before such a tool is used routinely in neonatal training programs across Canada, a need assessment is required to determine its current usage by accredited training programs. Our aim was to characterize the type of simulation modalities used and the perceived simulation-based training needs in Canadian neonatal-perinatal medicine (NPM) training programs.

Methods

A 22-item and 13-item online descriptive survey was sent to all NPM program directors and fellows in Canada, respectively. The survey was modeled on a previously validated tool by Johnston, et al. and responses were collected over 30 days.

Results

In total, eight (63%) program directors and 24 (28%) fellows completed the survey, with all respondents indicating that simulation is being used. Both lab-based and in situ simulations are occurring, with a range of simulation modalities employed to primarily teach resuscitation, procedural and communication skills. Fellows indicated that simulation should also be used to also teach other important topics, including disease-specific management, crisis resource management, and prevention of medical error. Five (63%) programs have faculty with formal simulation training and four (50%) programs have at least one faculty involved in simulation research.

Conclusion

Simulation is widely used in Canadian NPM training programs, with program directors and fellows identifying this as an important tool. Simulation can be used to teach a range of skills, but programs need to align their curriculum with both training objectives and learner needs. There is an opportunity for faculty development and increased simulation research.

## Introduction

Neonatal-perinatal medicine (NPM) is a high-acuity specialty, requiring efficient clinical-decision making skills and proficiency in complex procedural skills. This can make training in this field challenging and stressful. Furthermore, there are decreasing opportunities for trainees to gain competency in important skills, such as in emergency airway management and intubation, which ultimately affects physician competency and possibly patient safety [1-2]. This decrease in opportunities is multifactorial and related to issues such as decreased physician working hours, increased trainee numbers, and evolving management strategies [3].^ ^Therefore, given the decreased training opportunities along with patient safety concerns, there is now a paradigm shift from the traditional model of ‘see one, do one, teach one’ in the real-life situation to using simulation to gain experience and competency through deliberate practice [4-5].

Simulation is being widely used in a number of specialties, such as anesthesia, obstetrics, and emergency medicine. A recent survey by Doughty, et al. found that 95% of pediatric emergency medicine fellowship programs in the U.S. incorporate simulation-based training, with the remainder of programs planning to do so in the near future [6]. However, limitations were noted with respect to funding, space, equipment, and faculty.

In 2013, Eppich, et al. conducted a survey of both Canadian and U.S. pediatric emergency training programs with respect to the use of high-technology simulators. Sixty-three percent of programs at that time used high-technology simulators, mostly to teach decision making and technical skills [7]. In general, all program directors that were surveyed indicated value and the need to incorporate simulation into their training programs if not already done.

Focusing specifically on NPM training, Johnston, et al. conducted a survey of U.S. NPM training programs and found that 81% of programs that responded utilized simulation in their training, with 86% using ‘high-technology’ tools [8]. Simulation was used to teach a number of competencies such as resuscitation and procedural skills, crisis resource management, and professionalism. The amount of time training through simulation-based learning varied among the different programs. Barriers to the implementation of simulation-based training again included space, time, and cost [8]. In Canada, there are 13 Royal College of Physicians and Surgeons (RCPSC) accredited NPM training programs, which are two years in length. However, the use of simulation in these training programs has not been previously described in detail.

There is a clear trend towards the increased use of simulation in medical education. It is effective in improving knowledge and in many fields has been clearly associated with improved performance [9-10]. Simulation is being used for interprofessional training [11], and specialized simulation-based fellowship programs are expanding across North America [12]. With the shift towards competency-based curriculums, simulation is becoming increasingly important as a training and evaluation tool. Current faculty are indicating that they require simulation training to ensure effective teaching [13] and simulation is being considered not only for delivery of education but also for assessment and future accreditation [14]. However, as with any educational tool, its use should be based on clearly defined objectives and the most effective training modality should be adopted to meet those objectives. There can also be significant costs associated with the use of simulation-based training which warrant a cautious approach [15].

Therefore, with the increasing use of simulation-based training and the RCPSC mandated move towards competency-based curriculums, we conducted a study with the aim of characterizing the use of medical simulation along with perceived training needs in Canadian NPM training programs.

## Materials and methods

Study design

Two descriptive cross-sectional surveys were administered in this study. One survey was sent to all 13 NPM program directors in Canada, as identified by the RCPSC program director directory. A similar survey was sent at the same time to all NPM fellows enrolled in an accredited Canadian training program.

Survey tool

Two similar surveys were used for this study, a 22-item survey intended for NPM program directors (Appendix A) and a 13-item survey intended for fellows (Appendix B). Both program directors and fellows were asked to participate in order to compare their perspectives on simulation training. The survey sent to program directors also had more questions in order to explore issues such as faculty involvement and institutional support. These tools were adapted from a previously validated tool by Johnston, et al. [8], which was used to assess the use of medical simulation in NPM training programs in the United States. The survey tool was evaluated for content validity by the investigators and local experts in simulation.

Survey administration

The electronic surveys were administered online via Survey Monkey (Survey Monkey, 2016). All participants received an introductory letter along with a link to the appropriate survey via e-mail. Program directors received an e-mail directly from the study team and were asked to disseminate the survey to their trainees. All participants were given 31 days to complete the survey, with reminder e-mails sent at 14 and 21 days after the initial e-mail.

Research ethics board approval was obtained from the University of Toronto. Informed consent for participation was implied based on completion of the survey and no incentives were offered for participation.

## Results

In total, eight out of 13 (63%) program directors and 24 out of 66 (28%) fellows completed the survey. Of the program directors who responded, two led programs with less than five trainees, four led programs with five to 10 trainees, and two led programs with greater than 10 trainees. The surveys can be found in Appendix A (program directors) and Appendix B (fellows), where the questions have been grouped by category similar to the results and discussion below. Appendix C specifically identifies which questions in both surveys relate to the categories discussed below.

Simulation use

All respondents indicated that simulation was being used in their training programs. Low-technology mannequins were used in all programs, with high-technology mannequins and task trainers each being used in six out of eight (75%) programs, standardized patients in five out of eight (63%) programs, and one program reporting the use of fruits and meat as task trainers. No programs reported using screen-based or other virtual reality simulators.

Equipment and space

Half of the responding programs conduct both in situ and lab-based simulations, with three out of eight (38%) and one out of eight (13%) of the remaining programs conducting lab-based and in situ simulations alone respectively. Five out of eight (63%) programs reported operating a dedicated space for simulation, and remaining programs report having access to other local space for simulation. All programs owned at least a low-technology mannequin, with four out of eight (50%) programs owning a high-technology mannequin and five out of eight (63%) programs owning task-based trainers.

Curriculum

**Figure 1:**
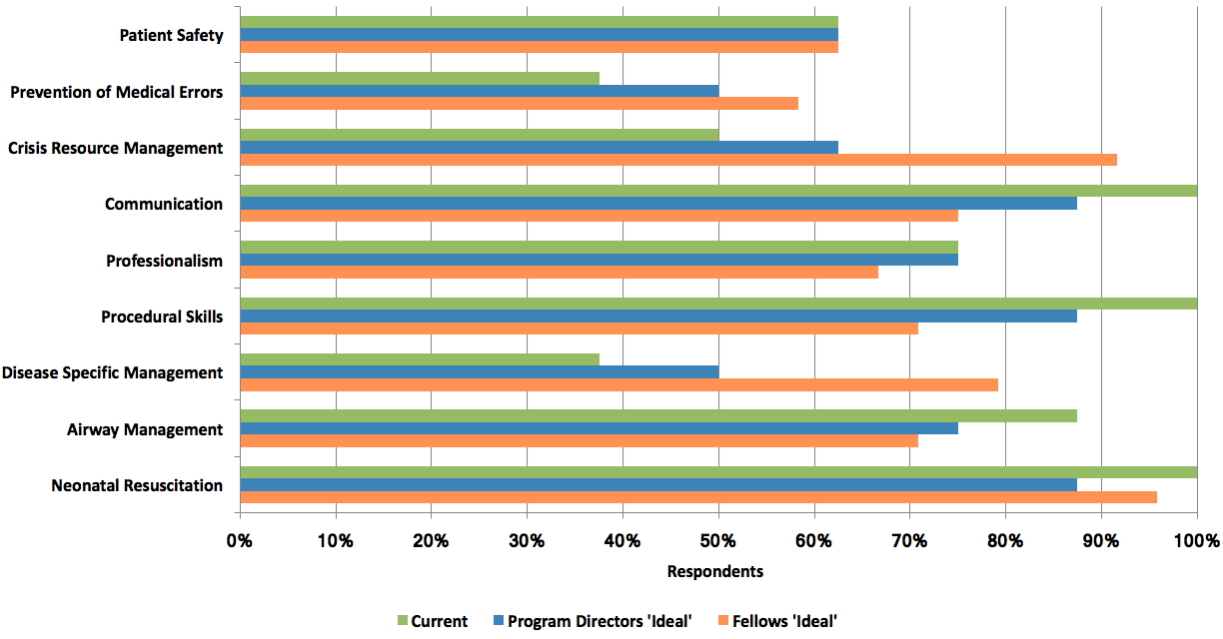
Objectives of Training

Four out of eight (50%) programs report having a fully developed curriculum that is in use. Other programs report to be developing (two out of eight - 25%) or revising (one out of eight - 13%) their curriculum, with one program (13%) reporting that they do not have a formal curriculum. Figure 1 shows the current objectives of simulation-based training along with topics that both program directors and fellows believe should be ideally taught using simulation.

Video debriefing is being used by four out of eight (50%) programs. Five out of eight (63%) programs report that simulation is being used for formative evaluation, with no programs using simulation for summative evaluation.

**Figure 2:**
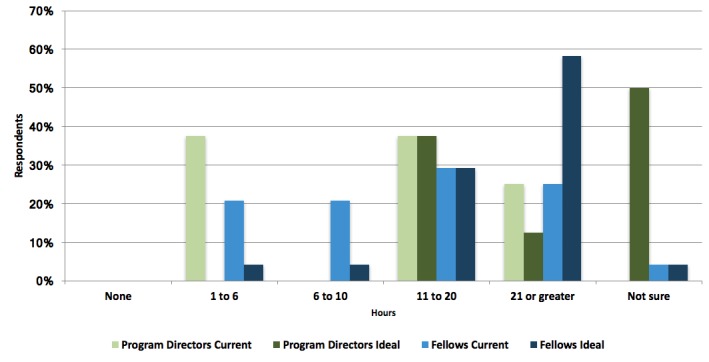
Hours of Simulation Training If a colored bar is not present in a specific group of hours along the x-axis, this indicates that the corresponding group did not choose that particular response.

The current number of hours of simulation training being offered as well as what program directors and fellows feel are ideal is shown in Figure 2.

Faculty

With respect to formal simulation training, five out of eight (63%) programs report having at least one faculty with such training. All programs have at least one faculty involved in designing cases/evaluation tools, with three out of eight (38%) programs having up to four faculty involved. Three out of seven (43%) programs report that faculty do not receive any specific recognition for teaching through simulation, with the remaining faculty receiving recognition towards tenure/promotion or monetary compensation. In terms of research, four out of seven (57%) programs report having one faculty and one program (14%) reported having three faculty involved in simulation related research.

The greatest barrier for faculty with respect to simulation training is time, which was identified by all programs, followed by lack of faculty training (38%), cost of equipment/operations (38%), lack of support staff (13%) and lack of access to learners (13%).

## Discussion

This study provides information on the current use of simulation in NPM training programs in Canada. This data helps to understand how simulation is being used, the perceived needs and barriers to its implementation and how it may be used as training programs move towards competency-based training and assessment.

Simulation use/equipment and space

All programs reported the use of simulation-based education and low-technology mannequins, while 75% of programs also used high-technology mannequins. The use of high-technology simulators is slightly less than that reported by Johnston, et al. (75% vs. 86%) in 2012 in US training programs, but the use of low-technology simulators is greater (100% vs. 58%) [8].^ ^This may reflect the increased use of simulation over time in NPM training programs, with low-technology mannequins being more readily accessible and less costly. It is important to note that low-technology simulators can be just as effective as high-technology simulators for learning [16]. The focus should be on the educational objectives and using the modality that will best meet those needs.

Curriculum

Our results indicate that there is a gap between some of the objectives of current simulation programs and the perceived needs of trainees. As reported by program directors, most NPM programs use simulation to teach neonatal resuscitation, procedural skills, airway management, and communication. These are the same objectives that program directors feel should be ideally taught through simulation. However, when trainees were asked what they feel simulation should ideally be used to teach, they identified crisis-resource management (CRM) skills, disease-specific management, and prevention of medical errors as being important, in addition to neonatal resuscitation and procedural skills. With the exception of neonatal resuscitation, the most common trainee-identified topics are currently taught by the least number of programs and identified as less important by program directors (Figure 1). The current focus of most programs on technical skills reflects what simulation has traditionally been used to teach [6-8], which is reasonable given the need for deliberate practice, decreasing clinical exposure, and the need to enhance patient safety. However, simulation is also being used successfully to teach non-procedural skills [17]. Therefore, there is an opportunity to expand the use of simulation in Canadian training programs to enhance the acquisition of non-technical skills and address the perceived needs of our learners.

At present, simulation is being used for formative evaluation as reported by 63% of programs. No programs are using simulation for summative evaluation, but as competency-based curriculums are becoming mandated, simulation may have a valuable role and this needs to be further explored. Recently, the Canadian National Anaesthesiology Simulation Curriculum (CanNASC) Task Force published their approach for the development and implementation of a simulation-based curriculum that all anaesthesia residents will need to satisfactorily complete to obtain certification [18]. This highlights the increasing use of simulation in competency-based education and evaluation.

There is a general trend of wanting more hours of simulation training among both program directors and trainees when asked how many hours of training would be ‘ideal’ (Figure 2). Interestingly, 50% of program directors identified that they were not sure how many hours would be ‘ideal’. This reflects the difficulty in defining the number of hours of simulation training that NPM programs should provide. The hours required likely vary based on the skill being learned and the learner. With the drive towards competency-based education in Canadian training programs, it will be important to better define how simulation can be used to help individual learners achieve competency in a variety of domains. For example, Doglioni, et al. calculated that a learner would need to perform 100 intubations to become proficient [19]._ _Simulation may be useful as performing such a number solely in the clinical environment during a defined training period can be difficult. Although there may be limitations with respect to fidelity, simulation does offer the opportunity for deliberate practice by providing trainees with a readily available tool and consistent experience [5]. However, the number of hours required to become proficient will be affected by learner time, cases seen, and opportunities for simulated practice. This also has implications for faculty, as learners not only need practice but also feedback in order to improve their skills [20]. Overall, there is a need to better support program directors, especially as programs begin designing and implementing competency-based curriculums.

In this study, we asked one question about debriefing and found that 50% of programs currently use video debriefing. Debriefing is a critical component of a simulation session [21]. In subsequent studies, there is an opportunity to further explore the specific educational techniques being used and understand teacher/learner perspectives and implications of such.

Faculty

Most programs (63%) have faculty who are formally trained in simulation-based education, and all programs have at least one faculty involved in designing cases or evaluation tools for the simulation programs. Unfortunately, 43% of programs report that their faculty do not receive any recognition for their involvement in simulation. Of note, the majority of programs (71%) report having faculty involved in simulation-based education research, with most programs (57%) having one faculty involved. Although this is encouraging, there appears to be ample opportunity for additional faculty to be involved.

The greatest reported barrier to the use of simulation training was time, followed by lack of faculty training, cost of equipment/operations, lack of support staff, and lack of access to learners. Although not asked in this study, other barriers such as being in a stressful/intimidating environment, fear of educator or peer judgment, and fear of inaccurate reflection of clinical ability have also been reported in the literature and may be important factors to consider [22]. One strategy may be to facilitate a shift in culture, in that as simulation and debriefing is used more commonly it becomes the accepted and expected standard to support learning. Given the similarities identified in both Canadian and US programs, there may be an opportunity to form new collaborations to address the identified barriers, especially with the drive towards new competency-based curriculums.

Limitations

Our study does have limitations. In our study, 63% (8/13) of NPM program directors responded but only 28% (24/86) of fellows responded to the survey. These rates are similar to survey response rates reported by Johnston, et al. [8] of program directors (62%) and Sawyer, et al. [23] of NPM fellows (22%). At present, there is no contact list that includes all current NPM trainees in Canada. Program directors whom we contacted do not have such a list, and there is no such accessible list from the RCPSC or Canadian Residency Matching service. Therefore, program directors have traditionally been asked to forward surveys to their trainees, which can result in unintended sample bias. Strategies to contact trainees directly, such as at national exams, exit surveys, or creating a national contact list, may be considered by the RCPSC, program directors, and education researchers to ensure that trainees are aware of such studies which could potentially lead to increased response rates. Unfortunately, with a low response rate, it is difficult to know whether we have obtained a representative sample of trainees across multiple programs. A higher response rate with the strategies outlined above would help to resolve this issue.

## Conclusions

Simulation is widely used in Canadian NPM training programs, with both program directors and fellows identifying it as an important tool. Simulation can be used to teach a range of skills, but programs need to align their training objectives with learner needs. There is an opportunity to further support faculty development in simulation-based education and to increase simulation education research in Canadian programs. This data provides a snapshot of simulation-based education in Canadian NPM programs. It is possible that similar themes would emerge in other pediatric training programs and this should be further explored. Much work remains to be done in defining how simulation can be most effectively used to teach and assess learners, especially as training programs move towards competency-based curriculums.

## Appendices

Appendix A: Survey – For neonatal-perinatal medicine program directors

1. How many neonatal-perinatal fellows are enrolled in your program each year?

( ) <5

( ) 5-10

( ) >10

Simulation use/equipment and space

2. Do your neonatal-perinatal medicine fellows use simulation as part of their training?

(i.e., this may include low or high-fidelity mannequins, task trainers, interactive computer software, or standardized patients)

( ) Yes

( ) No

3. If yes, where do simulations occur?

( ) Classroom or simulation center

( ) In situ (actual clinical space)

( ) Both

4. What type of simulators do your neonatal-perinatal fellows use? (Please check all that apply)

( ) Mannequin-based low-fidelity simulation (e.g., simple mannequin)

( ) Mannequin-based high-fidelity simulation (e.g., computer controlled)

( ) Task trainers (e.g., intubation trainer)

( ) Screen-based simulation (e.g., interactive case-based computer software)

( ) Standardized patients

( ) Other (please specify)

5. Which of the following does your division OWN? (Please check all that apply)

( ) One or more mannequin-based low-fidelity simulators

( ) One or more mannequin-based high-fidelity simulators

( ) One or more task-based simulators

( ) One or more screen-based simulators

( ) None

( ) Other (please specify)

6. Does your neonatal-perinatal fellowship program own/operate a SPACE dedicated to simulation training (e.g., medical simulation center)?

( ) Yes

( ) No

7. If the neonatal-perinatal fellowship program/division does not own simulation equipment or space dedicated to simulation training, do your fellows receive simulation-based training in another location within the institution or medical school?

( ) Yes

( ) No

8. If the neonatal-perinatal fellowship program/division does not own simulation equipment or space dedicated to simulation training, which entity is primarily responsible for the simulation equipment and/or space used by your fellows?

( ) Pediatrics Department

( ) Anesthesia Department

( ) Hospital

( ) Medical school

( ) Don’t know

( ) Other (please specify)

9. What type(s) of high-fidelity infant simulator mannequin(s) do your neonatal-perinatal fellows use? (please check all that apply)

( ) Laerdal SimBaby

( ) Laerdal SimNewB

( ) Gaumard Newborn HAL

( ) Gaumard Premie HAL

( ) Don’t know

( ) None

( ) Other (please specify)

Curriculum

10. If fellows participate in simulation training, the curriculum is designed to teach what? (please check all that apply)

( ) Neonatal resuscitation skills

( ) Airway management

( ) Disease-specific management (e.g., ductal dependent

cardiac lesion)

( ) Procedural skills

( ) Professionalism

( ) Communication skills

( ) Crisis resource management/team training

( ) Prevention of medical errors

( ) Patient safety

( ) N/A

( ) Other (please specify)

11. If fellows participate in simulation training, how many hours of training do they receive, on average, per year?

( ) None

( ) 1–6 hours

( ) 6–10 hours

( ) 11–20 hours

( ) 21 or greater hours

( ) Not sure

12. If fellows participate in simulation training, is video debriefing used?

( ) Yes

( ) No

( ) Not applicable/not sure

13. What barriers to using simulation exist within your program? (please check all that apply)

( ) Faculty time

( ) Lack of Neonatology faculty training in simulation

( ) Cost of equipment and operations

( ) Lack of support staff for the center/program

( ) Lack of access to learners

( ) None

( ) Other (please specify)

14. How would you describe your curriculum for simulation in neonatal-perinatal medicine?

( ) No curriculum

( ) Initial development

( ) Fully developed but NOT implemented

( ) Fully developed AND implemented

( ) Currently being revised

15. Is simulation being used as an assessment tool in your training program? If yes, how is it being used?

( ) Not being used for evaluation

( ) Formative evaluations

( ) Summative evaluations

16. Do you think that a simulation curriculum is important for your training program?

( ) Yes

( ) No

Why?

17. Ideally, how much time do you think that fellows should spend in simulation training, on average, per year?

( ) None

( ) 1–6 hours

( ) 6–10 hours

( ) 11–20 hours

( ) 21 or greater hours

( ) Not sure

18. What do you think are the most important skills that fellows should learn from simulation training? (please check all that apply)

( ) Neonatal resuscitation skills

( ) Airway management

( ) Disease-specific management (e.g., ductal dependent

cardiac lesion)

( ) Procedural skills

( ) Professionalism

( ) Communication skills

( ) Crisis resource management/team training

( ) Prevention of medical errors

( ) Patient safety

( ) N/A

( ) Other (please specify)

Faculty

19. Do faculty who teach through simulation have formal simulation training?

( ) Yes

( ) No

( ) Do not know

If yes, where was their training obtained?

20. How many of your neonatal-perinatal fellowship program faculty are involved with designing cases/evaluation tools for your human simulation curriculum?

( ) None

( ) 1

( ) 2

( ) 3

( ) 4

( ) >4

( ) Do not know

21. How many of your neonatal-perinatal fellowship program faculty are involved in education research in human simulation?

( ) None

( ) 1

( ) 2

( ) 3

( ) 4

( ) >4

( ) Do not know

22. What recognition do your neonatal-perinatal fellowship program faculty receive for participation in teaching/assessment with mannequin-based high-fidelity simulators? (check all that apply)

( ) None

( ) Release time

( ) Monetary compensation

( ) Recognition for tenure/promotion

( ) Do not know

( ) Other (please specify)

Please provide any additional comments.

Appendix B: Survey – For neonatal-perinatal medicine trainees

Simulation use

1. As a neonatal-perinatal medicine fellow, do you use simulation as part of your training?

 (i.e., this may include low- or high-fidelity mannequins, task trainers, interactive computer software, or standardized patients)

( ) Yes

( ) No

Equipment and space

2. If yes, where do simulations occur?

( ) Classroom or simulation center

( ) In situ (actual clinical space)

( ) Both

3. What type of simulators do you use? (Please check all that apply)

( ) Mannequin-based low-fidelity simulation (e.g.,

simple mannequin)

( ) Mannequin-based high-fidelity simulation (e.g.,

computer controlled)

( ) Task trainers (e.g., intubation trainer)

( ) Screen-based simulation (e.g., interactive case-based

computer software)

( ) Standardized patients

( ) Other (please specify)

4. Which of the following does your division OWN? (check all that apply)

( ) One or more mannequin-based low-fidelity simulators

( ) One or more mannequin-based high-fidelity simulators

( ) One or more task-based simulators

( ) One or more screen-based simulators

( ) None

( ) Other (please specify)

5. If the neonatal-perinatal fellowship program/division does not own simulation equipment or space dedicated to simulation training, do you receive simulation-based training in another location within the institution or medical school?

( ) Yes

( ) No

6. If the neonatal-perinatal fellowship program/division does not own simulation equipment or space dedicated to simulation training, which entity is primarily responsible for the simulation equipment and/or space used by fellows?

( ) Pediatrics Department

( ) Anesthesia Department

( ) Hospital

( ) Medical school

( ) Don’t know

( ) Other (please specify)

7. What type(s) of high-fidelity infant simulator mannequin(s) do you use? (please check all that apply)

( ) Laerdal SimBaby

( ) Laerdal SimNewB

( ) Gaumard Newborn HAL

( ) Gaumard Premie HAL

( ) Don’t know

( ) None

( ) Other (please specify)

Curriculum

8. If you participate in simulation training, the curriculum is designed to teach what? (please check all that apply)

( ) Neonatal resuscitation skills

( ) Airway management

( ) Disease-specific management (e.g., ductal dependent

cardiac lesion)

( ) Procedural skills

( ) Professionalism

( ) Communication skills

( ) Crisis resource management/team training

( ) Prevention of medical errors

( ) Patient safety

( ) N/A

( ) Other (please specify)

9. If you participate in simulation training, how many hours of training do you receive, on average, per year?

( ) None

( ) 1–6 hours

( ) 6–10 hours

( ) 11–20 hours

( ) 21 or greater hours

( ) Not sure

10. If you participate in simulation training, is video debriefing used?

( ) Yes

( ) No

( ) Not applicable/not sure

11. Do you think that a simulation curriculum is important for your training program?

( ) Yes

( ) No

Why?

12. Ideally, how much time do you think that fellows should spend in simulation training, on average, per year?

( ) None

( ) 1–6 hours

( ) 6–10 hours

( ) 11–20 hours

( ) 21 or greater hours

( ) Not sure

13. What do you think are the most important skills that fellows should learn from simulation training? (please check all that apply)

( ) Neonatal resuscitation skills

( ) Airway management

( ) Disease-specific management (e.g., ductal dependent

cardiac lesion)

( ) Procedural skills

( ) Professionalism

( ) Communication skills

( ) Crisis resource management/team training

( ) Error avoidance

( ) N/A

( ) Other (please specify)

Please provide any additional comments.

Appendix C: Categories and specific questions

Simulation use/equipment and space

Program Director Survey - Questions #: 2-9

Fellows Survey - Questions #: 1-7

Curriculum

Program Director Survey - Questions #: 10-18

Fellows Survey - Questions #: 8-13

Faculty

Program Director Survey - Questions #: 19-22

Fellows Survey - Questions #: no questions
